# Design of Hybrid Beamforming System Based on Practical Circuit Parameter of 6-Bit Millimeter-Wave Phase Shifters

**DOI:** 10.3390/mi14040875

**Published:** 2023-04-19

**Authors:** Mohammed A. Alqaisei, Abdel-Fattah A. Sheta, Ibrahim Elshafiey, Majid Altamimi

**Affiliations:** Electrical Engineering Department, King Saud University, Riyadh 11421, Saudi Arabia

**Keywords:** millimeter-wave (mm-wave), hybrid beamforming, phase shifter, 45 nm CMOS technology, silicon on insulator (SOI), single-pole double-throw (SPDT)

## Abstract

This paper addresses the design of a hybrid beamforming system considering the circuit parameter of six-bit millimeter-wave phase shifters based on the process design kit. The phase shifter design adopts 45 nm CMOS silicon on insulator (SOI) technology at 28-GHz. Various circuit topologies are utilized, and in particular, a design is presented based on switched LC components, connected in a cascode manner. The phase shifter configuration is connected in a cascading manner to get the 6-bit phase controls. Six different phase shifters are obtained, which are 180°, 90°, 45°, 22.5°, 11.25°, and 5.6°, with a minimum number of LC components. The circuit parameters of the designed phase shifters are then incorporated in a simulation model of hybrid beamforming for a multiuser MIMO system. The number of OFDM data symbols used in the simulation is ten for eight users, 16 QAM modulation schemes, −25 dB SNR, 120 simulation runs, and around 170 h runtime. Simulation results are obtained considering four and eight users, assuming accurate technology-based models of RFIC components of the phase shifter as well as ideal phase shifter parameters. The results indicate that the performance of the multiuser MIMO system is affected by the accuracy level of the phase shifter RF component models. The outcomes also reveal the performance tradeoff based on user data streams and the number of BS antennas. By optimizing the amount of parallel data streams per user, higher data transmission rates are achieved, while maintaining acceptable error vector magnitude (EVM) values. In addition, stochastic analysis is conducted to investigate the distribution of the RMS EVM. The outcomes show that the best fitting of RMS EVM distribution of the actual and ideal phase shifters agreed with the log-logistic and logistic distributions, respectively. The obtained (mean, variance) values of the actual phase shifters based on accurate library models are (46.997, 481.36), and for ideal components the values are (36.47, 10.44).

## 1. Introduction

Millimeter-wave approaches have proven to be an applicable and promising way to overcome the challenges and limitations of the lower frequency bands. The three most promising mm-wave bands are 28 GHz, 60 GHz, and the E-band, which address the capacity issue for the lower frequency limitation systems [1,2,3,4]. The 5G and beyond new radio (NR) wireless communication systems combine spatial multiplexing and massive multi-input multi-output (mMIMO) beamforming to increase data throughput at millimeter-wave (mm-wave) frequencies and achieve greater SNRs. The mm-wave and mMIMO technologies have recently appeared as probable solutions for improving the system capacity, data rate, and coverage of future mobile networks [5,6,7,8,9,10].

Path loss is significantly higher at the mm-wave range. Furthermore, as compared to ordinary radio frequency (RF) circuits, mm-wave is costly, lossy, and has high power consumption. To alleviate these limitations, beamforming is usually required at both the transmitter and the receiver [11,12,13,14]. In mm-wave massive MIMO, large-scale antenna arrays can be deployed due to the small wavelength of mm-wave signals and can be used to expand beamforming gain and synthesize directional beams [15,16,17].

Three main categories of beamforming technology have been investigated in the literature: digital (adaptive), analog (switched), and hybrid beamformer. In digital beamforming, one dedicated RF chain per antenna element is required [18,19]. On the other hand, various hardware components are required in large-scale antennas to enable digital beamforming, including mixers, power amplifiers, and digital-to-analog and analog-to-digital converters. Because of that, a significant number of hardware components are required for a digital beamforming system, which is undesirable in terms of both cost and power consumption, especially in mobile terminals and mm-wave frequency bands [17,20]. In analog beamforming, the antenna loads can either be applied using time-delay elements or phase shifter networks before or after the RF chain stage [21,22]. On the other hand, analog beamforming can only favor a single stream transmission and so cannot fully utilize the specific spatial resource [17].

Hybrid beamforming (HBF) that combines digital and analog beamforming is widely used in modern communication systems [17]. Analog beamforming is achieved using phase shifters to provide the directivity gains, while the multiplexing gains are provided using digital beamforming. System cost can be decreased considerably in hybrid beamforming by utilizing fewer RF chains to provide spatial multiplexing. In comparison with analog beamforming, hybrid beamforming provides multi-stream transmission with spatial division multiple access (SDMA) and spatial multiplexing (SM). In addition, it has good spectral efficiency and low hardware complexity comparable with digital beamforming [23,24,25,26,27,28,29].

A single-user hybrid beamforming approach in mm-wave bands was first presented in [30]. After that, hybrid beamforming in mm-wave was developed for either multiuser single carrier (MU-SC) [31,32,33] systems or single-user multicarrier (SU-MC) systems [34,35,36]. The essential objective of the hybrid beamforming approach is to reach high system performance using little RF chains and the high-dimensional phase shifter [17,37,38]. In mm-wave mMIMO communication systems, a hybrid beamforming architecture balances beamforming improvements and power consumption, as well as hardware cost. In massive MIMO systems, a popular rule of thumb is that the ratio between the number of antennas to the number of users should be more than ten, such that user channels are orthogonal and give optimal efficiency. It is feasible to minimize computational complexity in hybrid beamforming for mm-wave communication systems by using fewer analog RF chains (in comparison to the number of users), and their performance is comparable to that of optimum digital beamformers [39].

Typical analysis of the performance of HBF depends on the theoretical model of analog phase shifters [10,39]. The accurate design depends on the model of phase shifters representing the used RFIC. This should consider both the used technology as well as the actual process design kit from the IC manufacturer. Taking into consideration accurate models of the RF components of the phase shifter provides a design of the hybrid beamforming system that accounts for RF impairments, which is referred to as dirty RF [40]. The developed model thus allows the assessment of the level of impact of the analog phase shifter and design of the digital beamforming component to cope and mitigate the impact of analog RF components on system performance [41]. Mitigating RF imperfections associated with dirty RF is also referred to as green radio, which includes green design and green transmission [42].

Recently, phase shifters used in modern communication systems have been based on RFIC technologies, such as 0.18 um SiGe, 0.13 um SiGe, 28 nm CMOS, 28 nm LP-RF CMOS 1P7M, and 45 nm CMOS SOI [43].

We base this research on SOI technology using a 45 nm CMOS process from GlobalFoundries (GF). The process design kit (PDK) is made available to the researchers based on an agreement with GF. The devices created with silicon-on-insulator (SOI) structures are not the same as those made with bulk silicon. SOI configuration consists of a silicon layer on top of electrical insulators such as SiO_2_. The type of insulator material to choose is mostly determined by the application. The advantage of SOI is that it enables fabrication of devices with high speed, low power consumption, and radiation-hardening properties [44,45]. Phase shifters can be implemented using SOI technology [46,47].

The adopted technology is suitable for supporting mm-wave beamforming applications for the fifth generation (5G) communication systems. A CMOS SOI-based switched-LC phase shifter gives more linearity and reasonable loss, and thus this technology is appropriate for beamforming front-end modules (FEMs). The technology is also suitable for the back-end-of-line (BEOL) characteristics of broadband wireless systems to improve the performance for power amplifiers, LC-switches, and low noise amplifiers (LNAs) [48,49].

In this research, a 6-bit 28-GHz phase shifter in 45 nm CMOS SOI technology is developed and implemented in the analysis of hybrid beamforming systems. While various circuit topologies have been proposed, the developed phase shifter consists of a switched-LC component configuration. Six different phase shifts are obtained as 180°, 90°, 45°, 22.5°, 11.25°, and 5.6°. By considering the accurate models of the phase shifter components based on the process design library, more realistic hybrid beamforming models are obtained. Simulation results are presented for the root means square error vector magnitude (RMS EVM), and stochastic distribution is derived corresponding to the level of signal-to-noise ratio (SNR).

The remainder of this paper is organized as follows: Section 2 describes the design of the suggested phase shifter and hybrid beamforming. The results and discussions are discussed in Section 3. The conclusion is presented in Section 4.

## 2. Phase Shifter Circuit and Hybrid Beamforming Design

**Figure 1:**
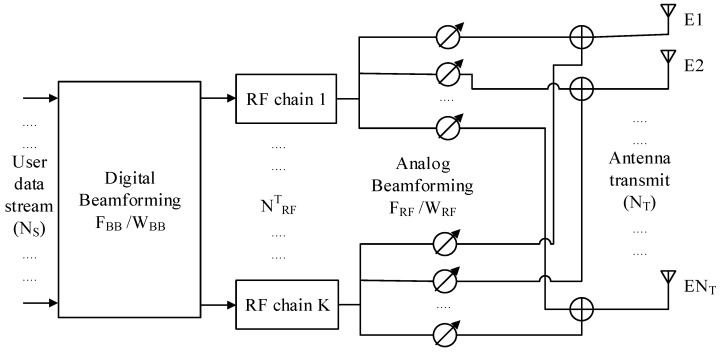
Hybrid beamforming for multi-user massive MIMO system at the transmitter.

The hybrid beamforming model for a multi-user massive MIMO system is shown in Figure 1. The trade-offs for cost, complexity, and flexibility determine the combining of the baseband digital domain with the RF analog domain [50,51]. Accomplishing beamforming in the baseband digital domain requires the use of the channel matrix to determine combining weights and precoding. This step can facilitate the independent transmission and recovery of multiple data streams across a single channel [30]. However, in the RF analog domain, beamforming is accomplished by using phase shifts to the antenna subarray’s antenna element [52].

Matlab environment is used to build this model by using the downlink for data transmitted from BS to multi-users. A scattering MIMO channel (3D multipath propagation channel) is considered in this model, in which radiated signals from a transmitting array are reflected from multiple scatters back toward a receiving array. A random collection of rays inside a 3-dimensional space is used to cover as many scatters as is practical [30]. The simulation model adopts an orthogonal matching pursuit technique for a single-user scenario. In the multi-user case, the joint spatial division-multiplexing algorithm is applied, and user groups are defined based on the channel covariance matrix. An analog predecessor based on the block diagonalization technique can be used to reduce inter-group interference [53].

Details of the simulation model are described in [51]. Simulation is conducted with ten OFDM data symbols. The M-ary QAM modulation scheme with m = 16, 64, and 256 is used. The sampling rate is set at 100 Mbps, and the channel code rate per user is 1/3. The noise figure is selected as 8 dB. The range of mobile stations from BS is taken to be less than 1000 m, and azimuth, as well as elevation angles, are chosen to obtain a 3D channel around BS.

The simulation considers a downlink with a uniform rectangular array at the transmitter (BS), and a uniform linear array at the MS receiver. The number of transmitter antenna-array elements are chosen to be 64, 128, and 256, while the number of array elements at the receiver depends on the number of streams. The number of users is chosen to be four and eight. An independent channel is allocated to every user. An RF chain is utilized to transmit an independent data stream.

The mobile stations are placed based on random distribution. For four user cases, the locations happened to be at distance values of 546, 859, 686, and 332 m. The azimuth angles are picked at −158.4°, −40.94°, −103.27°, and 155.7°, and the elevation angles at 40.11°, −81.56°, 57.84°, and 16.54°. On the other hand, for eight users, the mobile stations are set at 546, 859, 686, 332, 60, 387, 214, and 933 m. The azimuth angles in degrees are set as 80.22°, −163.12°, 115.67°, 33.07°, −89.25°, −66.57°, −112.38°, and −53.89° for each user, respectively. The user elevation angles in degrees are set at −43.7°, −87.99°, −16.27°, −15.79°, −82.23°, 81.91°, −18.42°, and 5.34°, respectively.

The simulation is conducted for a single data stream and multiple data streams. The number of independent multiple data streams for each user for four user cases is chosen as [2 1 3 2] and for the eight-user case is [3 2 1 2 1 2 3 2].

**Figure 2:**
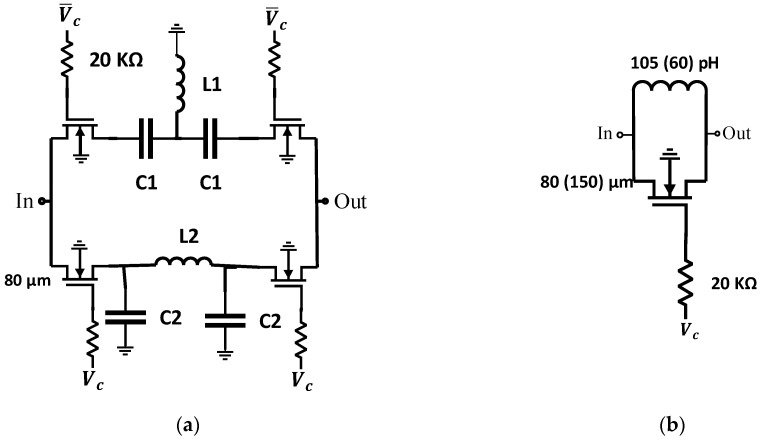
Schematics of (**a**) 180°, 90°, 45°, 22°, and (**b**) 11° (5°) phase shifters.

The main component of the transmit and receive module in an RF frontend beamforming is the phase shifter. The phase shifter can be designed using vector modulators or switched-LC components. However, the switched-LC phase shifter in 45 nm CMOS SOI offers higher linearity and a low loss when compared with the vector modulators [43,54,55]. Two different phase shifter topologies shown in Figure 2 are used in this paper to validate the concept. The first configuration is based on switching between low-pass (LP) and high-pass (HP) networks. Three passive elements arranged in π/T configuration are used for LP/HP, respectively [56].

The schematic of this network is shown in Figure 2a. For each phase shifter, switching between LP and high pass networks is achieved by two single pole dual through (SPDT) switches. The SPDT switch consists of two MOSFETs, developed based on 45 nm CMOS SOI technology. A MOSFET model of 80 µm width and 45 nm length is used in these four phase shifters. This configuration is used to design 22°, 45°, 90°, and 180° phase shifters. For a matching purpose, inductances and capacitances can be shown to be given by [56]:

(1) $$L_{1}=\frac{1}{\omega \sin\left(\frac{\Delta \emptyset }{2}\right)}\;C_{1}=\frac{1}{\omega \tan\left(\frac{\Delta \emptyset }{4}\right)}\;L_{2}=\frac{\tan\left(\frac{\Delta \emptyset }{4}\right)}{\omega }\;C_{2}=\frac{\sin\left(\frac{\Delta \emptyset }{2}\right)}{\omega }$$

where Δ∅ is the phase shift, and *L*_1_, *L*_2_, *C*_1_, and *C*_2_ are the inductors and capacitors shown in Figure 2a, normalized with respect to the transmission line impedance Z_0_ and admittance Y_0_, respectively. The configuration is optimized using Keysight Advanced Design System (ADS) simulator to improve the return loss to be less than 10 dB in the entire frequency band.

**Figure 3:**
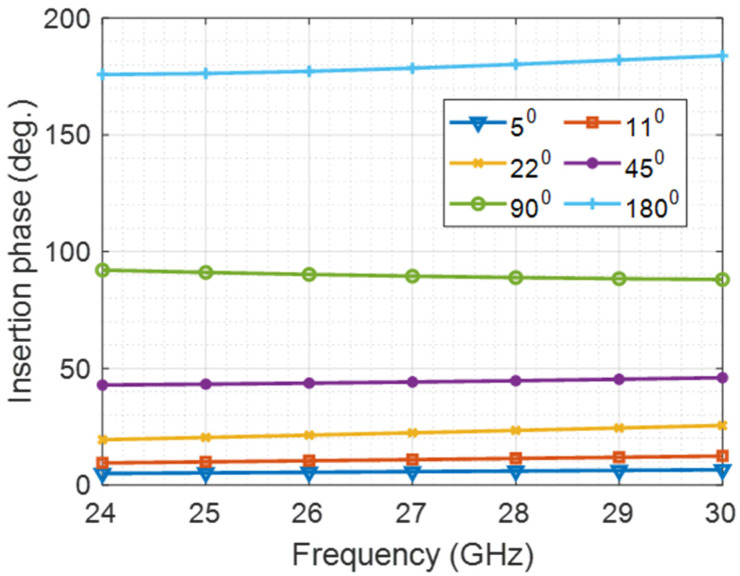
Simulated (180°, 90°, 45°, 22°, 11°, and 5°) phase shifters.

The values of the inductances and capacitances of each case are given in Table 1 for phase shifts of 180°, 90°, 45°and 22.5°. For 11.25° and 5.6° phase shifts, a different configuration is used with the schematic shown in Figure 2b. The structure consists of a MOSFET switch of width 150 µm for 5.6° and 80 µm for 11.25°, connected in parallel with an inductance of 60 pH for 5.6° and 105 pH for 11.25°. High voltage on the gate presents zero phase reference, and zero voltage on the gate presents the required phase in each case. The 6-bit phase shifters consist of four passive switched-LC component phase shifters beside two configurations of the circuit in Figure 2 connected in cascade to achieve one output of 64 possible phase shifts. 180°, 90°, 45°, 22.5° 11.25°, and 5.6° are the potential targets of phase shifters that meet valid 64 phase shifts. The lumped LC components and the transistor capacitances were optimized to get a phase shifter near the target. Figure 3 shows the scattering parameters of all cases of 180°, 90°, 45°, 22°, 11°, and 5° phase shifters.

**Figure 4:**
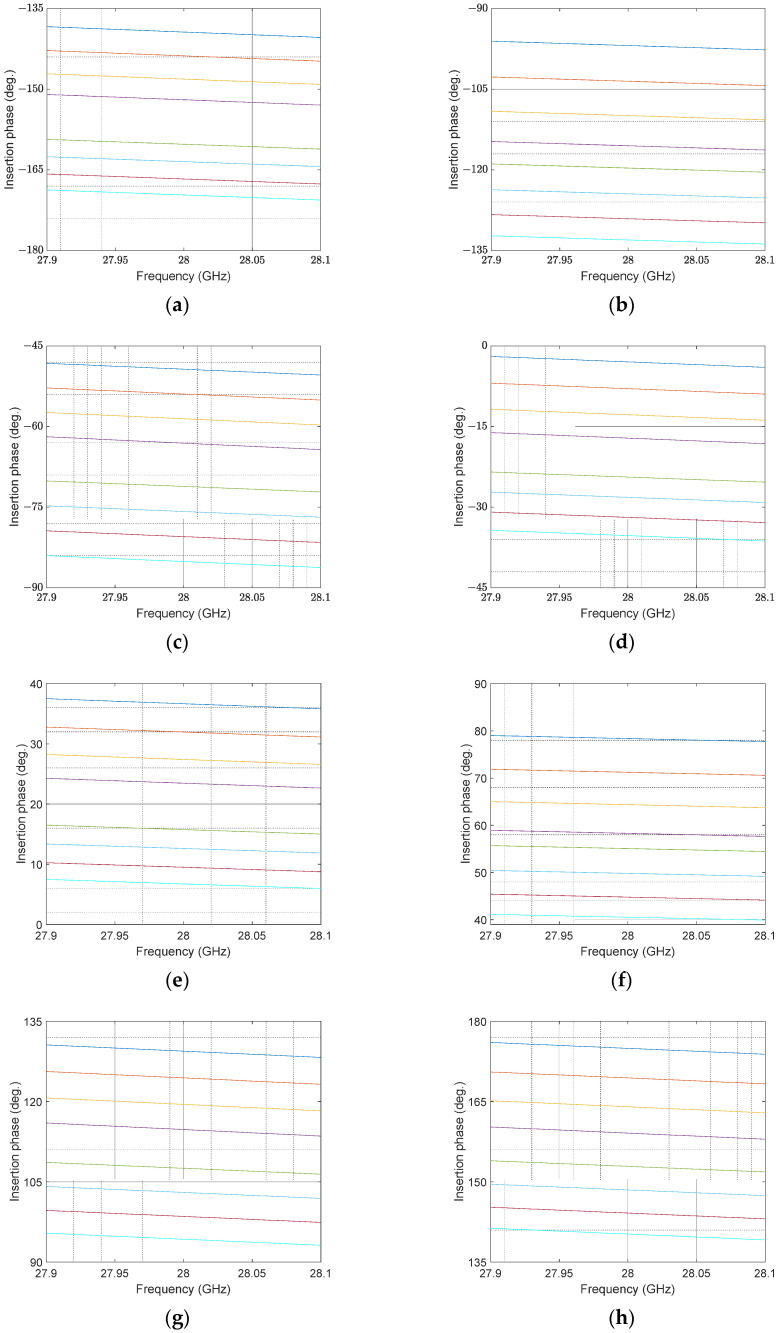
Simulated phase shifter in all 64 phase states for 6-bit phase shifters, corresponding to phase shift of: (**a**) −180°–−135° (**b**) −135°–−90° (**c**) −90°–−45° (**d**) −45°–0° (**e**) 0°–40° (**f**) 40°–90° (**g**) 90°–135° (**h**) 135°–180°. Different colors in each figure correspond to 8 different phase levels.

To perform hybrid beamforming, numerical scattering parameters are exported from the full-wave simulator and subsequently processed using the Phased Array System Toolbox in MATLAB. The proposed design alternates cascade passive switched-LC phase shifter cells to result in 6-bit phase control. The practical cascading of the designed 6-bit phase shifters will not be similar to an ideal phase shifter. The 64-phase states are then combined with our custom code to create analog beamformers based on a practical circuit phase shifter, as illustrated in Figure 4.

**Figure 5:**
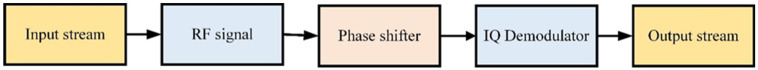
Modeling of actual phase shifter element.

Figure 5 shows the block diagram of the actual phase shifter used in a hybrid beamforming architecture. The RF signal converts the Simulink signal to the RF block set circuit envelope voltage or current. Here, the phase shifter replaces the S-parameters of the ideal phase shifter parameters with our actual phase shifter parameters. An IQ demodulator achieves perfect reconstruction of the in-phase component and quadrature component by exploiting the quadrature-phase relation between the signal in-phase component and a signal quadrature component.

## 3. Results and Discussions

**Figure 6:**
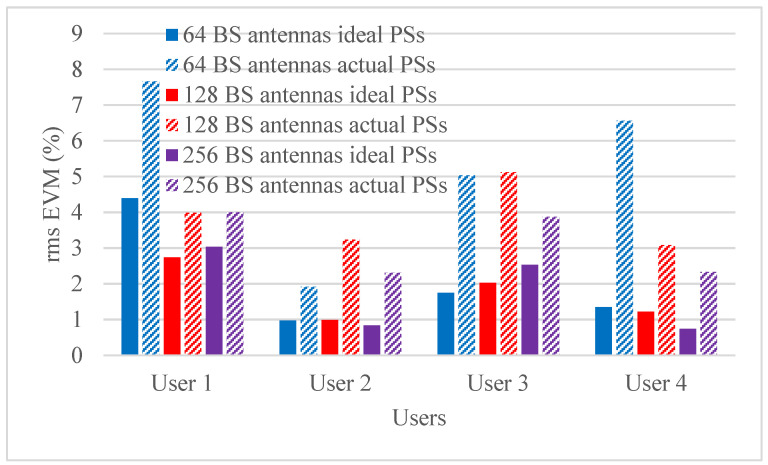
RMS EVM values of single data stream for actual and ideal phase shifters using the 16-QAM scheme and multiple BS antennas for four users when using four receiver antennas.

**Figure 7:**
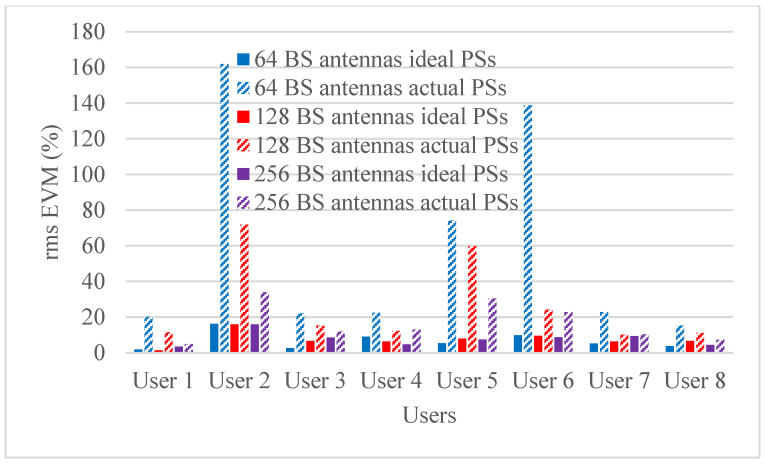
RMS EVM values of single data stream for actual and ideal phase shifters using the 16-QAM scheme and multiple BS antennas for eight users when using four receiver antennas.

**Figure 8:**
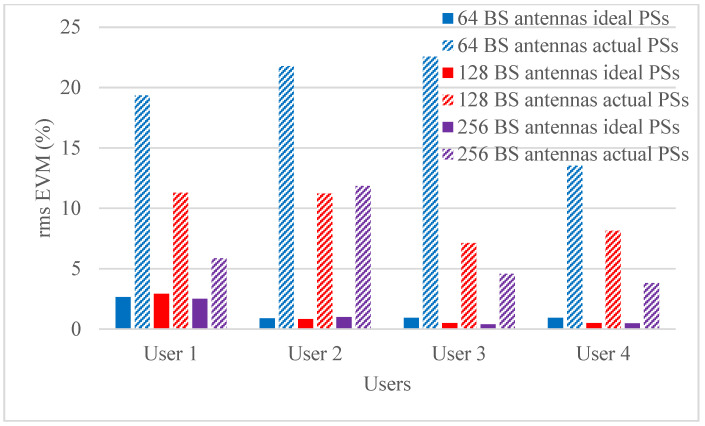
RMS EVM values of multiple data streams for actual and ideal phase shifters using a 16-QAM scheme and multiple BS antennas for four users when using four receiver antennas.

**Figure 9:**
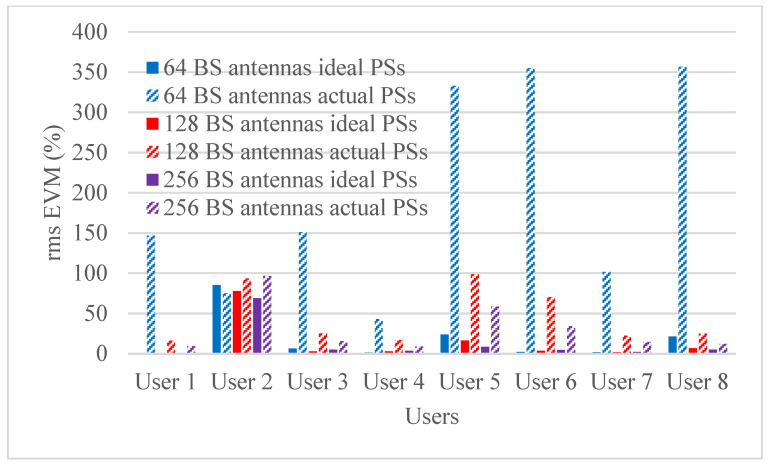
RMS EVM values of multiple data streams for actual and ideal phase shifters using a 16-QAM scheme and multiple BS antennas for eight users when using four receiver antennas.

Hybrid beamforming methods are established in multi-user massive MIMO systems to decrease training overhead and hardware costs at the mm-wave spectrum band. The designed phase shifters are implemented in the hybrid beamforming system and results are compared with ideal phase shifters. The RMS EVM values for both actual phase shifters and ideal phase shifters are shown in Figure 6, Figure 7, Figure 8 and Figure 9. Figure 6 and Figure 7 show RMS EVM for the 16-QAM modulation schemes for multi-user massive MIMO systems of four and eight users, respectively, with 64, 128, and 256 BS antennas for a single data stream. From the outcomes of Figure 6 and Figure 7, it is clear that RMS EVM for hardware model-based phase shifters is greater than that of ideal phase shifters. In addition, increasing the BS transmit antennas reduces the RMS EVM for both actual and ideal phase shifters. However, extending the number of users from four to eight also causes an increasing RMS EVM for both actual and ideal, as shown in Figure 6 and Figure 7. As expected, the RMS EVM performance depends also on the user’s location, azimuth angles, and elevation angles. For multiple data streams, Figure 8 and Figure 9 illustrate the RMS EVM values of the 16-QAM modulation scheme for multi-user massive MIMO systems with 64, 128, and 256 BS antennas for four and eight users, respectively. From the results of Figure 8 and Figure 9, it is concluded that RMS EVM for actual phase shifters is greater than that of ideal phase shifters. Moreover, increasing the BS transmit antennas minimizes the RMS EVM for both actual and ideal phase shifters. However, raising the number of users from four to eight also causes an increase in RMS EVM for both actual and ideal, as shown in Figure 8 and Figure 9.

**Figure 10:**
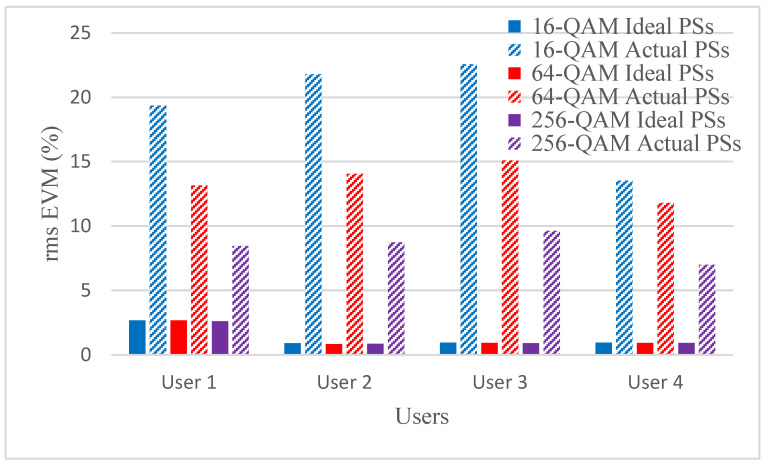
RMS EVM values of multiple data streams for actual and ideal phase shifters using M-QAM schemes and 64 BS antennas for four users when using four receiver antennas.

**Figure 11:**
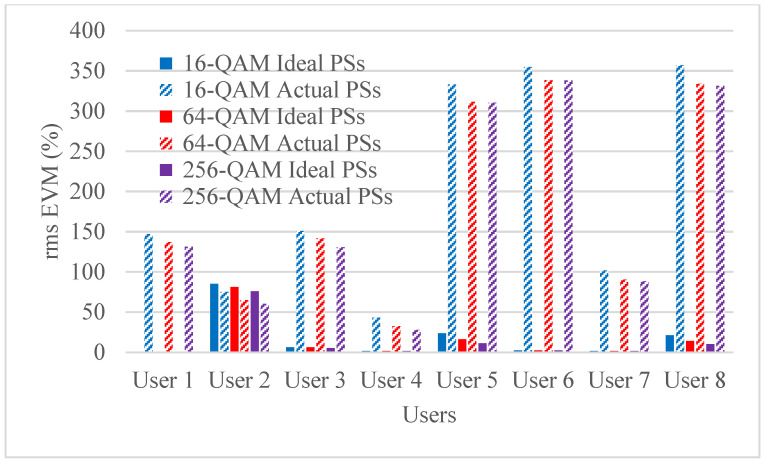
RMS EVM values of multiple data streams for actual and ideal phase shifters using M-QAM modulation schemes and 64 BS antennas for eight users when using four receiver antennas.

Figure 10 and Figure 11 illustrate the RMS EVM values of the 64 BS antennas for multi-user massive MIMO systems with various modulation schemes for four and eight users, respectively. It is noted from the results that for the four users, the RMS EVM performance improves significantly when the number of M-QAM modulation schemes is increased in the actual phase shifters. However, the RMS EVM is slightly improved for the ideal phase shifters. Similar behavior is noted for eight users with slightly improved RMS EVM. In general, increasing the number of M-QAM modulation schemes improved the RMS EVM performance.

**Figure 12:**
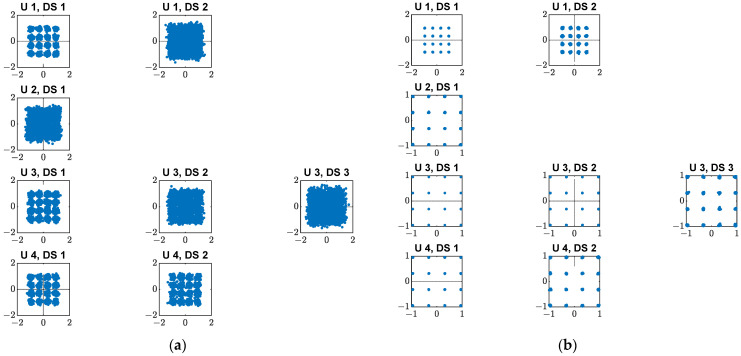
64 BS antennas with equalized symbol constellation per data stream for four users of the 16-QAM modulation scheme: (**a**) Actual phase shifter; (**b**) Ideal phase shifter.

**Figure 13:**
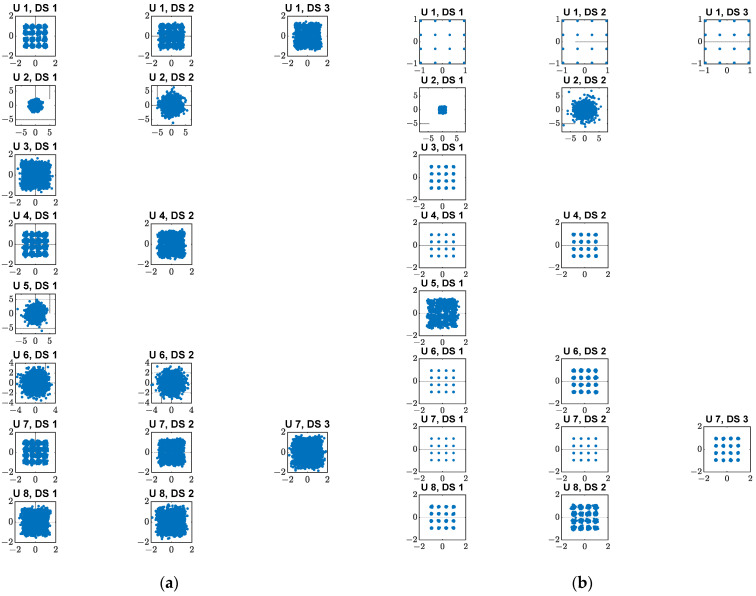
64 BS antennas with equalized symbol constellation per data stream for eight users of the 16-QAM modulation scheme: (**a**) Actual phase shifter; (**b**) Ideal phase shifter.

The equalized symbol constellations per data stream for both actual and ideal phase shifters with 64 BS antennas for four users with multiple data streams for the 16-QAM modulation scheme are depicted in Figure 12a,b, respectively. Figure 13a,b illustrate the equalized symbol constellation for both actual and ideal phase shifters with 64 BS antennas for eight users with multiple data streams for the 16-QAM modulation scheme, respectively. It can be noticed that for the same user, the variation of the recovered data streams is high for the actual phase shifter compared with the ideal phase shifter.

The RMS EVM distributions are a critical measure of performance for multi-user massive MIMO hybrid beamforming systems. They are impacted by the number of users, modulation scheme, mobile stations’ locations, and other factors. Here, we investigate the distributions of RMS EVM for ideal and actual phase shifters. The number of OFDM data symbols used in this simulation is ten for eight users, 16 QAM modulation schemes, −25 dB SNR, 120 simulation runs, and 170 h runtime. Investigating the obtained results, it is found that the actual RMS EVM distribution is different from that of the ideal RMS EVM. Table 2 shows the log-likelihood of different distribution types. It is clear that the minimum log-likelihood of the actual RMS EVM distribution is the log-logistic distribution, whereas the logistic for both ideal RMS EVM distribution is the difference between actual and ideal.

**Figure 14:**
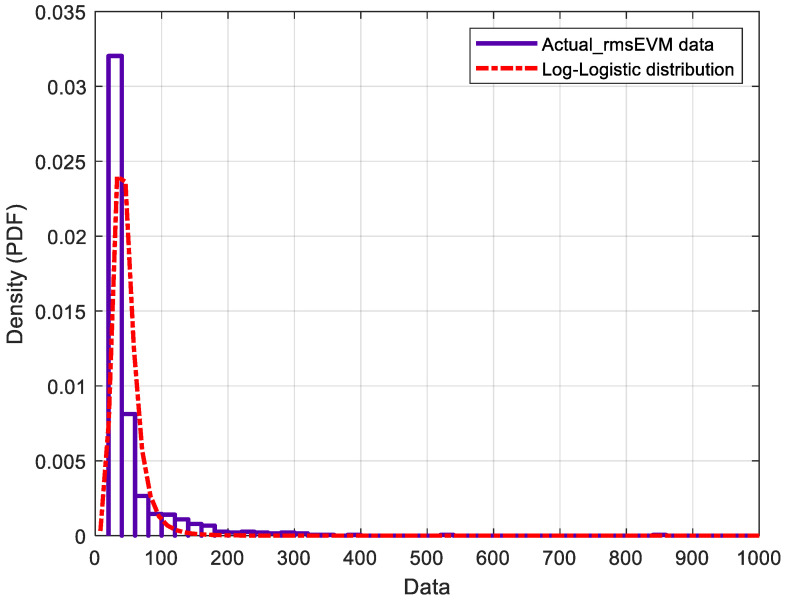
Fitting of the probability distribution function of an RMS EVM distribution using an actual phase shifter.

**Figure 15:**
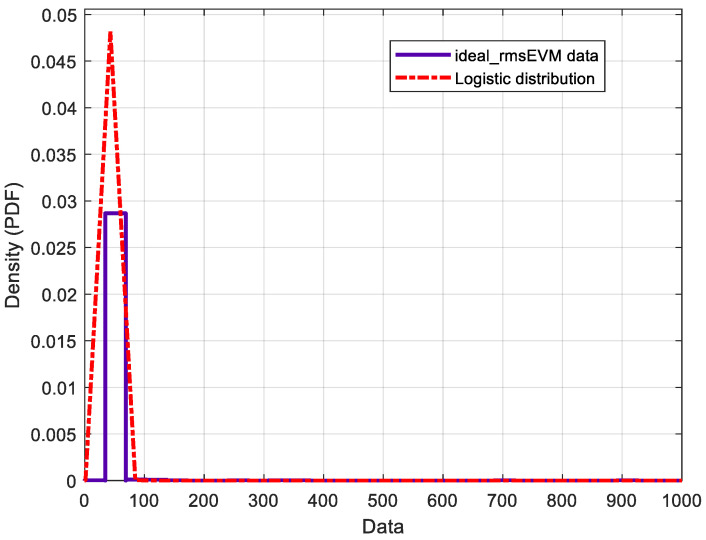
Fitting of the probability distribution function of an RMS EVM distribution for an ideal phase shifter.

Figure 14 shows that the RMS EVM distribution of the actual phase shifter agrees with the log-logistic theoretical distribution. The log-logistic distribution, on the other hand, is produced by applying the logarithmic transformation to the logistic distribution in the same way, such that the log-normal distribution is produced from the normal distribution. The ideal RMS EVM distribution is also similar to the theoretical distribution of logistics shown in Figure 15. The logistic distribution is utilized in different growth models, as well as in some regression schemes. The standard logistic distribution represents a continuous distribution on R, with distribution function G given by [57]

(2) $$G\left(z\right)=\frac{e^{z}}{1+e^{z}},z\in \mathbb{R}$$

**Figure 16:**
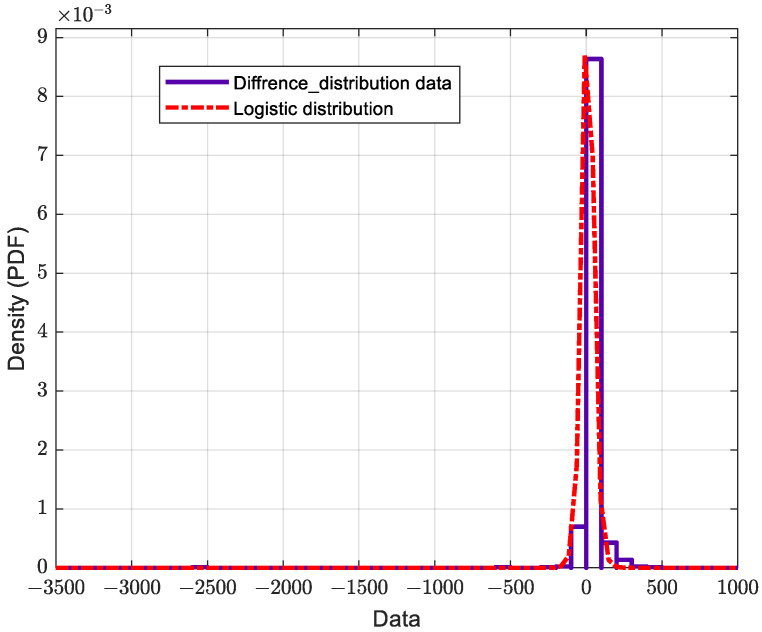
The probability distribution function of an RMS EVM distribution of the difference between actual and ideal phase shifters.

The difference between these two distributions is shown in Figure 16. It is clear that this difference represents a logistic distribution. The mean and variance of the actual phase shifters are 46.997 and 481.359 respectively, whereas the mean and variance of the ideal phase shifters are 36.4697, and 10.4387 respectively. Table 3 shows the other parameterizations of the estimated distributions with more details. In this table, µ refers to mean for logistic distribution and to mean of logarithmic values for log-logistic distribution.

## 4. Conclusions

This paper presents a 6-bit 28-GHz phase shifter using a 45 nm CMOS SOI technology for a multi-user massive MIMO hybrid beamforming system. The parameters of the phase shifters are adopted in a simulation of the hybrid beamforming system, where the simulation parameters used in this work are ten OFDM data symbols used for eight users, 16 QAM modulation schemes, −25 dB SNR, 120 simulation runs, and about 170 h runtime.

The developed phase shifters are based on the optimization of the lumped LC components and the transistor capacitances using a full-wave EM simulator. The designed phase shifter provides high linearity and reasonable loss. In this paper, the single and multiple data stream users are explored. The number of antennas for each user is chosen to be four times the number of streams to be received by this user. The spatial diversity results in a reduction in the RMS EVM with the increase of the data streams for the same user. In addition, increasing the BS transmit antennas reduces the RMS EVM for both actual and ideal phase shifters. Therefore, extending the number of users from four to eight is found to increase RMS EVM for both actual and ideal model analyses.

The results show that the RMS EVM for phase shifters based on accurate models of RF components based on the process is greater than that of ideal phase shifters. This indicates the importance of conducting an analysis of the beamforming system based on an actual model of analog phase shifters. Moreover, the RMS EVM performance improves significantly when the number of M-QAM modulation schemes is increased for the actual phase shifters. However, for the ideal phase shifters, the RMS EVM is slightly improved. It is noticed that for the same user, the variation of the recovered data streams is higher for the actual phase shifter than for the ideal phase shifter.

To characterize the difference between analyses using actual phase shifters compared to ideal phase shifters, a distribution is obtained for the impact of phase shifters on the distribution of the RMS EVM for multi-user massive MIMO hybrid beamforming systems. The estimated RMS EVM distribution of the actual and ideal phase shifters follow the log-logistic and logistic distributions, respectively. Furthermore, the estimated mean and variance parameters are 46.997 and 481.359, respectively, for the actual phase shifter, and 36.4697 and 10.4387, respectively, for the ideal phase shifter. Accounting for the actual behavior of the analog phase shifter based on accurate RFIC models allows coping and mitigating of RF impairments, which introduces the paradigm of green radio design in hybrid beamforming systems.

## Figures and Tables

**Table 1:** Optimized inductor and capacitor values of the configuration of the phase shifter in Figure 2a.

Phase Degrees	L1 (pH)	L2 (pH)	C1 (fF)	C2 (fF)
180°	284	384	125	80
90°	223	157	500	5.5
45°	6200	246	1680	38.8
22.5°	1783	46.9	1227	40.8

**Table 2:** Log-likelihood of different distribution types.

Distribution Type	RMS EVM for Actual Model of Phase Shifters	RMS EVM for Ideal Phase Shifters	Difference between Actual and Ideal
Exponential	−4877.62	−4650.74	----------
Log-Logistic	−4317.32	−2756.11	----------
Logistic	−4920.82	−2344.26	−5151.58
Nakagami	−5011.59	−5341.06	----------
Normal	−5401.58	−6232.33	−6240.35

**Table 3:** This is different parameterizations of the different distributions.

	Distribution Type	Log-Likelihood	Mean	Variance	Parameter Estimate Standard Error			
					µ	µ Error	σ	σ-Error
RMS EVM for Actual Model	Log-Logistic	−4317.32	46.997	481.359	3.7622	0.01226	0.2291	0.006769
RMS EVM for Ideal Model	Logistic	−2756.11	36.4697	10.4387	3.5926	0.00235	0.04862	0.001503
Difference between actual and ideal	Logistic	−5151.58	10.9149	1852.58	10.915	1.18592	23.7301	0.719523

## Data Availability

Not applicable.
